# Polymeric nanoparticle-based electrochemical sensor for the detection of CA 125

**DOI:** 10.55730/1300-0527.3524

**Published:** 2022-11-02

**Authors:** Ceren TÜRKCAN, Sinan AKGÖL

**Affiliations:** 1 Departmet of Biomedical Engineering, Faculty of Engineering and Architecture, İstanbul Arel University, İstanbul, Turkey; 2 Department of Biochemistry, Faculty of Science, Ege University, İzmir, Turkey

**Keywords:** Cys-graft-p(HEMA) nanomaterials, CA 125, electrochemical determination, point of care (POC)

## Abstract

In this paper, Cys-graft-p(HEMA) nanomaterials and a new electrochemical method were developed for determination of CA 125. Cys-graft-p(HEMA) nanomaterials were synthesized with emulsion polymerization method and modified with grafting procedure. It was determined that Cys-graft-p(HEMA) nanomaterials had 50 nm dimension and spherical morphology, and per gram polymeric material contained 0.011 mmol L-cysteine. Electrode surface was prepared step by step for electrochemical analysis with optimization process. Linear determination range was determined as 5–400 U/mL (R= 0.9935). Detection limit (LOD) was calculated as 1.87 U/mL, and quantification limit (LOQ) was determined as 5.62 U/mL. The fabricated sensor system showed good repeatability, accuracy, reality, and storage stability. According to the results obtained, Cys-graft p(HEMA) nanomaterials that is used for the first time in biosensor has the potential to find use in the sector with rapid determination time (10 min), extensive determination range, accuracy of methods. Novelties of this study are rapid analysis, determination range, appropriate of prototype device development, and developing new designed material. Developed material and method can be used in the preliminary diagnosis of the disease and combined with a prototype device that can allow the follow-up of the treatment process in diagnosed patients.

## 1. Introduction

Ovarian cancer is the fourth most frequent malignancy in women around the world. It is the most common type of cancer that causes death among female genital organ cancer types. Each year, 220,000 women are diagnosed with ovarian cancer [1]. The high mortality rate is due to the lack of early detection. A difficult period begins for patients diagnosed at an advanced stage [2]. The disease can reach the third stage in as little as 18 months [1]. The disease manifests itself with persistent groin and abdominal pain, as well as swelling around the abdomen. In addition to these symptoms, eating difficulties, feeling full quickly, and the need to urinate frequently are among the symptoms. Since the symptoms are not specific, they can often be confused with intestinal problems. As the disease progresses in people with these symptoms (Stage III or IV), the chance of treatment decreases. A staged ovarian cancer has a 95% chance of being serous. It is a very aggressive type of cancer [3,4].

Being able to be synthesized in different forms offers a wide range of advantages to polymeric materials. Polymeric nanomaterials have a wide use in the medical field, especially thanks to the interest in nanomaterials that are becoming more and more widespread today for their surface area and prosperity [5]. In addition to the diagnosis and treatment of diseases, it is widely used in imaging techniques or removal of some chemicals from body fluids [6–8]. In addition to the areas of use described with examples, polymeric nanomaterials also find use in the field of diagnostics. In the diagnosis of many diseases, in addition to the use of polymeric nanomaterials by modifying the target analyte to provide affinity, molecular imprinted polymers also allow selective and specific recognition because they contain specific wells for the target analyte [9,10]. Cysteine containing polymeric nanoparticle is used for attaching onto gold surface using sulfur of cysteine as seen in schematic presentation of the literature and manuscript [11,12]. The grafting procedure has been preferred in many studies because it offers numerous possibilities and diversity. When a new material design is desired, it is preferred to create a suitable chemical environment for ligand, receptor, or mediator molecules to bind [13,14]. In this study, as in other studies, cysteine-grafted polymeric nanoparticles were preferred in order to interact with the antibody and bind to the gold electrode surface and avoid the ligand leakage problem [15,16]. On the other side, nanoparticles are preferred in sensor studies due to the positive parameters such as the sensitivity limit they offer due to their large surface area and the possibility of determination in a wide scale [17].

In the literature, cancer antigen 125, a biomarker utilized in the diagnosis and follow-up of ovarian cancer, is also known as carbohydrate antigen 125 [8,18]. In the case of ovarian cancer, the level of CA 125 in the blood might be crucial. Values below 35 U/mL CA 125 indicate people who do not have ovarian cancer. CA 125 values above 35 U/mL mostly refer to people with ovarian cancer. The progression in the disease stages or the size of the mass is directly related to the amount in the blood. Therefore, it is a biomarker with high sensitivity and specificity [19,20]. For this reason, there are studies in the literature using nanomaterials for the electrochemical determination of CA 125. In this paper, Cys-graft-p(HEMA) nanomaterial was developed for electrochemical determination of CA 125. Cys-graft-p(HEMA) nanomaterial was characterized physically and chemically with 5 different methods. Electrochemical method was optimized for analysis of CA 125 linearly. Detection limit (LOD) was determined as 1.87 U/mL quantification limit (LOQ) was determined as 5.62 U/mL. After the experiment and calculating, assays were repeated 5 times with double repeat for repeatability experiments. The results were determined in the degree of accuracy within the scope of statistical calculations according to the precision and accuracy tests. In accordance with obtained results, these methods are thought to be used as alternative methods to applied CA 125 determining methods nowadays according to rapid determination time, extensive determination range, accuracy of methods.

## 2. Materials and methods

### 2.1. Materials

Ethylene glycol dimethacrylate (EGDMA), 2-hydroxyethylmethacrylate (HEMA), potassium persulfate (KPS), poly(vinyl alcohol) (PVA), L-cysteine, morpholinoethanesulfonic acid (MES), N-hydroxysuccinimide (NHS), N-(3-dimethylaminopropyl)-N′-ethylcarbodiimide hydrochloride (EDC), potassium chloride (KCI), bovine serum albumin (BSA), sulfuric acid (H_2_SO_4_), and hydrogen proxide (H_2_O_2_) were obtained from Sigma-Aldrich (Germany). Cancer antigen 125 (5kU) and anticancer antigen were obtained from Fitzgerald (USA).

Potassium hexacyanoferrate (III)-(II) K_3_[Fe(CN)_6_]/K_4_[Fe(CN)_6_] and potassium dihydrogen phosphate (KH_2_PO_4_) were obtained from Merck.

### 2.2. Methods

#### 2.2.1. Synthesis of polymeric nanomaterials

P(HEMA) nanomaterials were synthesized with emulsion polymerization methods. Polyvinyl alcohol (PVA, d: 1.269 g/mL) 0.275 g was dissolved in 25 mL of distilled water as a stabilizer using a heater mixer. PVA solution was added on reactor, and 0.6 mL of HEMA (d: 1.074 g/mL) monomer and 0.3 mL of EGDMA (d: 1.051 g/mL) were added as cross linker. Next, 0.0198 g of KPS was dissolved in 45 mL of water that has been distilled using a magnetic stirrer and added onto reactor. The mixture was treated with nitrogen gas and shaking water bath used for polymerization at 70 °C (shaking rate was 100 rpm). After 1 h, polymerization period finished and polymer solution was centrifuged at 14,100 × *g* for 20 min. P(HEMA) nanomaterials were washed 2 times with ethanol and 2 times with distilled water (99.8%). Finally, p(HEMA) nanomaterials were dried in an incubator at 37 °C [21–26].

#### 2.2.2. Grafting procedure

Twenty grams of p(HEMA) nanomaterials was added to the reactor, 1.4 g of NaH was added onto dry polymer, and 3 g of L-Cys dissolved in 50 cm^3^ tetrahydrofuran was added onto the mixture.

**Figure 1:**
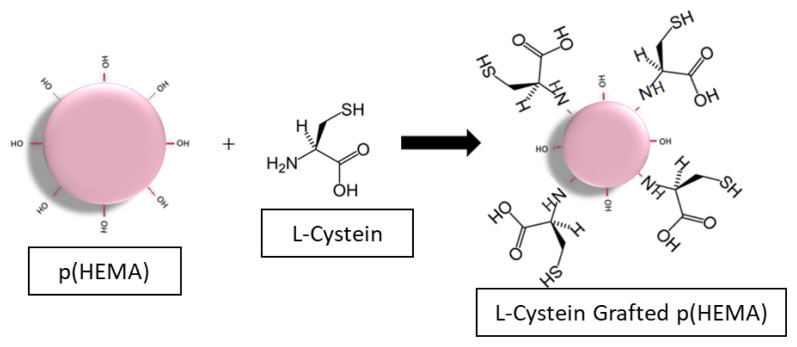
Schematic representation of the grafting procedure.

The grafting reaction was carried out by mixing this mixture at 40 °C for 24 h [27]. The same washing procedure was applied after 24 h and p (HEMA) nanomaterials were dried at 37 °C in an incubator [28]. In the grafting process, the bonding of the hydroxyl groups on the particle and the amino groups in the amino acid is ensured as seen in Figure 1.

After the grafting procedure, surface that can be modified was obtained and also L-cysteine containing polymeric nanoparticles can be attached onto gold surface.

#### 2.2.3. Characterization of cys-graft-p(HEMA) and p(HEMA) nanomaterials

FTIR, Zeta-Size, SEM, and elemental analyses and surface area calculation were done for characterization. FTIR analysis was performed to comment on the chemical composition of the polymeric nanomaterials, Zeta-Size analysis was performed to determine the size, SEM analysis to obtain morphological images, and elemental analysis to determine whether the L-cysteine amino acid was added to the p(HEMA) polymeric nanomaterial [5].

Specific surface area calculation method is below;
Surface area (m^2^/g) = N.SA/g polymer (dry mass)
N = 6 × 10^10^ × S / π × ρs × d^3^
N: 1 mL particle amount in the suspension
SA: Surface area of sphere (4.π.r^2^)
G polymer is per gram polymeric material.
r = Particle radius
S = 10 (dry mass %)
ps = Density of polymer
d = Particle radius (micrometer)
π = 3.14

#### 2.2.4. Preparation of sensor solution

For the preparation of sensor solution, 745 mg of KCl (0.1 M), 680.5 mg of KH_2_PO_4_, 211.2 mg of K_4_[Fe(CN)_6_], 165 mg of K_3_[Fe(CN)_6_] 5 mM (1:1) with precision balance were weighed and dissolved in 100 mL ultrapure water. The outer part was then covered with aluminum foil in an amber bottle and stored at room temperature [8].

#### 2.2.5. Preparation of sensor surface

By treating the gold electrode surface with a cysteine-grafted polymeric nanoparticle, interaction is established between the gold surface and the sulfur group in the amino acid. The surface is activated by EDC-NHS so that the amino group of the antibody can interact through the carboxyl group exposed in the amino acid. Antigen was attached with amino groups onto modified Cys-graft-p(HEMA) surface. After the activation process, the antibody is added to the surface and finally the surface is prepared to recognize the antigen by blocking with BSA.

**Figure 2:**
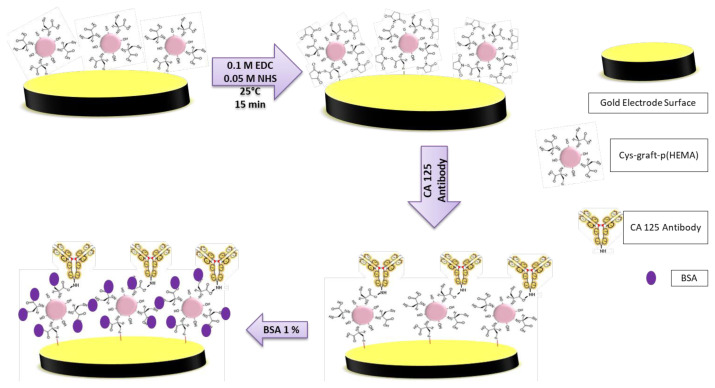
Schematic representation of sensor’s surface preparation for CA 125 determination.

Sensor surfaces were prepared step by step as follows. Alumina mixture was used for cleaning on gold electrode surface for about 15–30 min. Five microliters of Cys-graft-p(HEMA) nanomaterials (concentration is 4.55 mg/mL) were dropped onto gold electrode surface and kept at room temperature for 2 h. EDC and NHS were used for activation p(HEMA) nanomaterial to bonding CA 125 antibody at room temperature (15 min). Ten microliters of CA 125 antibody (concentration was 50 μg/mL) was dropped onto activated p(HEMA) nanomaterials at room temperature (1 h). Surface blocking was done with BSA (1 %) at room temperature (1 h). CA 125 was added onto surface of electrode (5 μL, 25 °C). There is a schematic presentation in Figure 2.

#### 2.2.6. Electrochemical analysis procedure

All electrochemical experiments were done using PalmSense system. Three electrode systems were used as analysis electrodes. Ag/AgCI electrode was the reference electrode and platinum wire was auxiliary electrode. Electrochemical analysis was taken between −0.4 and 0.8 V and 0.05 voltage rate was done in sensor solution in every seconds with 3 repetitions ([8,29]. The same criteria were applied in differential pulse voltammetry (DPV) and cycle voltammetry (CV).

#### 2.2.7. Electrochemical analysis of anti-CA 125 antibody

Polymeric nanomaterials were prepared for attaching to anti-CA 125 antibody on gold electrode surface. Five microliters of anti-CA 125 antibody was dropped onto electrode surface and kept for 10 min. Concentration ranges were 5, 10, 30, 60 100, 120, 150, 180, 250, 400, and 1000 U/mL. DPV and CV analyses were done with the same method, and peak current height was determined [30,31].

#### 2.2.8. Accuracy, reproducibility, storage stability

By assaying CA 125 levels in two sera for five replicate measurements using the same analytical procedure, the accuracy and repeatability parameters were assessed. Differential pulse voltammetry (DPV) and cyclic voltammetry (CV) were used to measure the electrodes that had been prepared in 0.1 M pH 7.4 phosphate buffer for 1 week to 20 days (CV). According to the measurement results, the electrode activity was compared with the previous data to determine whether there was any decrease in the electrode activity. Comparisons were made as a percentage (%) comparison over the amount of determinations obtained previously [32–34].

#### 2.2.9. Analysis in serum sample

ECLIA serum test standards, which are used as standard in hospitals for the determination of Ca 125, were used to test the sensor system. Standard serum samples determined in the ECLIA test as 30, 50, and 70 U/mL were analyzed with the developed method and the results were determined from the calibration chart.

## 3. Results and discussions

### 3.1. Characterization results of polymeric nanomaterials

**Figure 3:**
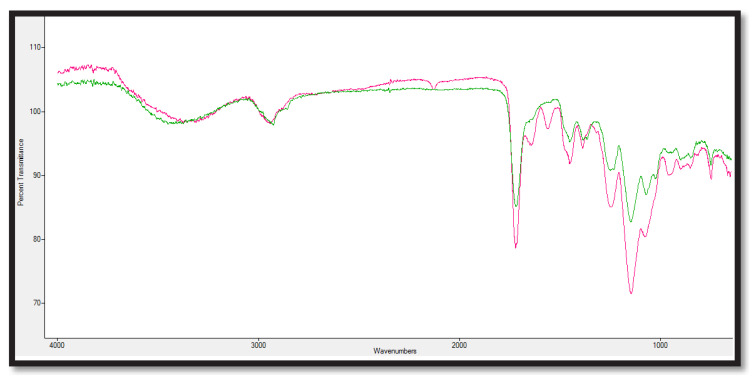
FTIR spectrum image of Cys-graft-p(HEMA) and p(HEMA) polymeric nanomaterials.

FTIR analysis was used to determine the structure of p(HEMA) polymeric nanomaterial synthesized using surfactant-free emulsion polymerization method and Cys-graft-p(HEMA) nanomaterial obtained after grafting process and used in all experiments. In Figure 3, the green spectrum is the p(HEMA) polymeric nanomaterial, and the pink spectrum is the FTIR spectrum of the Cys-graft-p(HEMA) nanomaterial.

As can be seen, the p(HEMA) nanopolymer has -OH bonds at 3500 cm^−1^, CH alkyl bonds at 3000 cm^−1^, C=O bonds at 1718 cm^−1^, and C=C bonds at 1500 cm^−1^. As seen in the pink spectrum at 1566 cm^−1^, the newly formed shoulders are thought to belong to the H-N bonds belonging to the L-cysteine amino acid added to the structure by grafting. It is predicted that there are vibrational bands belonging to the S-H group at 970 cm^−1^ and 1130 cm^−1^, the specific band thought to belong to the L-cysteine amino [21,35].

**Figure 4:**
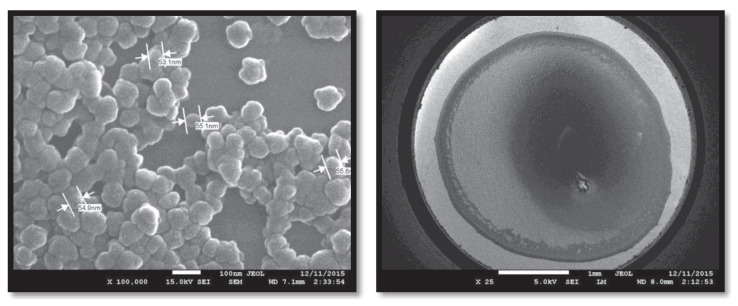
SEM images of Cys-graft-p(HEMA) nanomaterial on gold surface.

Cys-graft-p(HEMA) nanomaterials, which were synthesized and prepared by the grafting process, were examined with scanning electron microscope (SEM) photographs in order to visualize the surface morphology and general structure on both the glass surface and the gold surface. SEM photographs of the polymeric nanomaterial at different magnifications are shown in Figure 4 below.

The images obtained on the gold surface are the images obtained at 25× and 5000× magnifications. As seen in the images, the same and small size of the polymeric nanomaterial, which is also located on the gold electrode surface in this technique, suggests that the measurement range will be wide in antigen determination due to its high reproducibility and wide surface area due to its small size.

**Figure 5:**
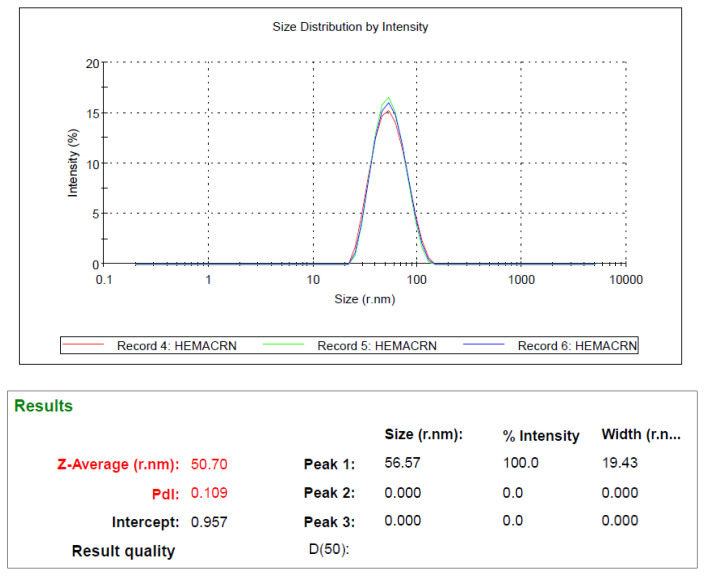
Zeta-Size analysis of quercetin imprinted polymeric nanoparticles.

The result of the dimensional analysis of the synthesized Cys-graft-p(HEMA) nanomaterial with Zeta-Size is shown in Figure 5 below.

The average dimensions of the synthesized Cys-graft-p(HEMA) nanomaterial are around 50.7 nm. As can be seen from the results of the analysis, it is seen that polymeric nanomaterials are obtained in very close sizes and their sizes are also quite small. According to the results, we can say that synthesized polymeric nanoparticles are monosized. It is of great importance that the polymeric materials used in sensor systems are equidimensional. Because the macro-sized interventions on very small surfaces and the preparation of the surfaces, the smallest differences in the small interventions made during the process as a result of the differences in the dimensions can cause maximum changes in the signals. Therefore, working with isodimensional polymeric nanomaterials makes coworking conditions even more possible in this sense.

**Figure 6:**
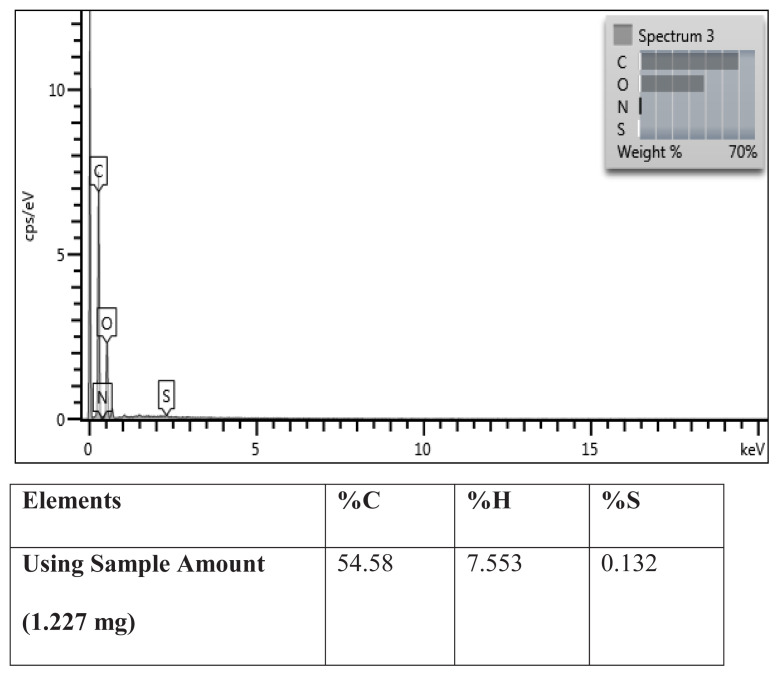
Elemental analysis result of Cys-graft-p(HEMA) nanomaterials.

Elemental analysis result of Cys-graft-p(HEMA) nanomaterial is shown in Figure 6 below.

Based on the results obtained as a result of elemental analysis, it can be said that L-cysteine amino acid is added to the structure of Cys-graft-p(HEMA) nanomaterial. The L-cysteine content of the Cys-graft-p(HEMA) nanomaterial was calculated based on the analysis result in Figure 6, the molecular mass of the cysteine amino acid, and the amount of sample used in the analysis. Considering that cysteine contains 1 sulfur atom and 1.227 mg of polymeric nanomaterial is used in elemental analysis, 0.132% of this polymeric nanomaterial is sulfur, so there is 0.0016 mg of sulfur in a 1.227-mg sample. (0.0016 mg = 0.0000016 g)

### 3.2. Results of electrochemical analysis

**Figure 7:**
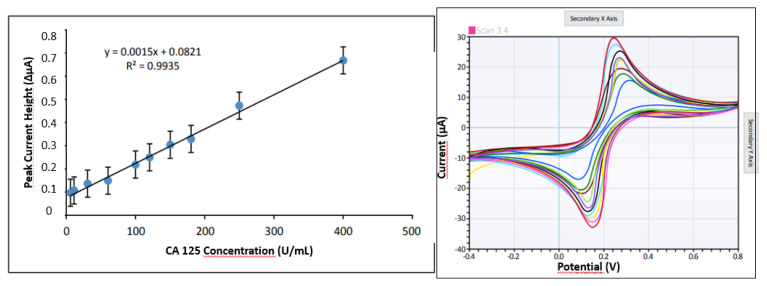
a) Results obtained by optimizing the amount of CA 125 determination. b) Concentration potential values were changed between −0.4 and 0.8 V. *(5 μL of 4.55 mg/mL Cys-graft-p(HEMA) nanomaterial with 2-h incubation at 25 °C, activation with EDC-NHS, 10μL of 50 μg/mL antibody at 25 °C for 1 h, Blocking with 1% BSA, 5 μL of antigen, 10 min at 25 °C, 5-10-30-60-100-120-150-180-250-400 U/mL antigen samples.)*

In order to optimize the determination of CA 125 amount, the delta peak current height values calculated by using the peak current heights in the voltammograms obtained as a result of the measurements against the CA 125 concentrations studied at 10 different concentrations are plotted in Figure 7a. Study voltammogram is as seen in Figure 7b.

When the findings are analyzed, the determination range can be stated linearly between 5 and 400 U/mL, detection limit (LOD) was obtained as 1.87 U/mL, and quantification limit (LOQ) was determined as 5.62 U/mL. It is seen that the determination range covers a very wide scale. Since 35 U/mL value, which is a critical value for ovarian cancer disease, is within the detection range, it is thought that healthy and suspected individuals can be accurately distinguished using this method. Considering the studies in the literature, if a study using SPR is shown as an example, the range of 0.1–40 U/mL is specified as the linear detection range [36]. In another study, using impedance spectroscopy, the detection range of CA 125 was specified as 0.1–30 U/mL [37]. In another method developed as an amperometric immunosensor, the detection range of CA 125 was specified as 2–75 U/mL [8]. When these studies are considered, in the light of the results obtained, it is thought that the detection range is wider than the studies in the literature. This allows the study to better distinguish between healthy and suspected patients. In addition to these, when we make a comparison in terms of cost, we can say that the developed polymeric nanomaterial-based sensor system is the one of the cheapest.

### 3.3. Accuracy, reproducibility, storage stability

**Figure 8:**
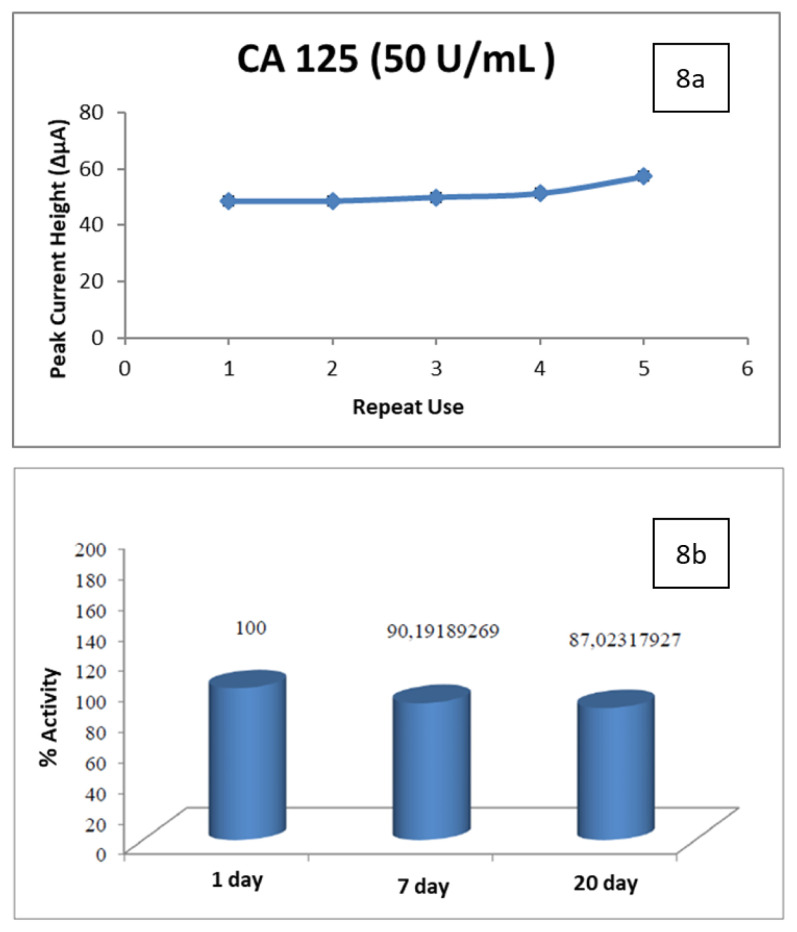
a) Reproducibility results. b) % values of activities obtained as a result of storage stability trials. *(5 μL of 4.55 mg/mL Cys-graft-p(HEMA) nanomaterial with 2-h incubation at 25 °C, activation with EDC-NHS, 10μL of 50 μg/mL antibody at 25°C for 1 h, blocking with 1% BSA, 5μL of 50 U/mL antigen, 10 min at 25 °C, 10 min*).

CA 125 concentrations, which were repeated as 50 U/mL, were obtained as 48.6 U/mL, 48.6 U/mL, 49.93 U/mL, 51.26 U/mL, and 57.26 U/mL, respectively, according to the measurement results as seen in Figures 8a and 8b. In the results of the storage stability trials, it was determined that the activity continued at the level of 90.2% and 87.0% for 7 days and 20 days, respectively.

Considering the result obtained, it is thought that the amount of activity lost by the prepared electrodes is low and will enable determination. In this way, thanks to the electrodes prepared in advance, the process time can be reduced to a short time like 10 min. It is thought that the determination of CA 125 can be made in a planned manner by preparing the electrodes to be used in the following days within the same day as the determinations are made on the one hand. According to the results obtained, the accuracy, reproducibility, and storage stability of the developed method are high.

### 3.4. Results of analysis in serum sample

**Figure 9:**
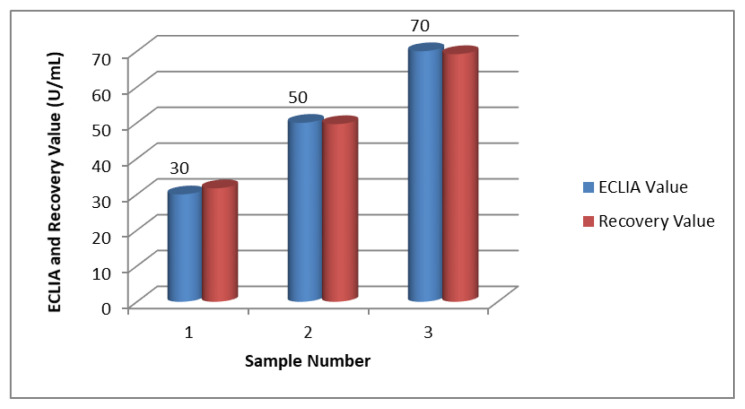
CA 125 amount was measured with ECLIA and developed methods. *(5μL of 4.55 mg/mL Cys-graft-p(HEMA) nanomaterial with 2-h incubation at 25 °C, activation with EDC-NHS, 10μL of 50 μg/mL antibody at 25 °C for 1 h, blocking with 1% BSA, 5μL of antigen, 10 min at 25 °C, 10 min*).

The following chart in Figure 9 shows the measurement results of serum test standards of 30, 50, and 70 U/mL with the developed method.

The values of the measurements (recovery value) whose results are given in the graph are given in Table 1 below.

The results obtained when the ECLIA test serum standards, which are routinely applied in CA 125 analysis in hospitals, are measured with the developed electrochemical method, are given in the table. Looking at the graph and the table, the results obtained are within the acceptable limits.

Table 2 below contains information about the methods and materials developed for CA 125 analysis developed in recent years.

When looking at the detection range and detection time in general, the method mentioned in the study appeals to a wider range than other methods and the measurement time is quite short. In the ECLIA test, which is used in hospitals apart from the literature, the measurement time is longer than the duration of the developed measurement method. In this regard, the developed method is advantageous because it gives fast results. Although the LOD value seems to be lower than other studies, it actually gives results well below the range that should be detected. Other studies appear to be more sensitive to LOD. However, according to the calibration graph obtained in the study, the analysis range is one of the widest studies. It is predicted that the developed method will analyze the healthy-diseased or risky group correctly. The developed material and method provides an advantage in terms of determination time and in terms of giving results in a very short time. In addition, the polymeric nanoparticles used in the study include cost-effective and completely domestic synthesis. The biggest disadvantage of the study is the length of time the sensor surface is prepared for measurement. After the study is completed, there is an idea to prepare polymeric nanoparticles and apply them in one step by making preliminary preparation by performing the operations without electrode.

## 4. Conclusions

In this study, Cys-graft-p(HEMA) nanomaterial was synthesized and characterized for the purpose of CA-125 determination. Cys-graft-p(HEMA) nanomaterial was not used before on sensor and gold surface. The characterized material is 50 nm in size and contains 0.0011 mmol L-cysteine. After the optimization studies, the determination range can be expressed linearly in the range of 5–400 U/mL, and detection limit (LOD) was calculated as 1.87 U/mL. It was determined that the activity continued as 90.2% after 7 days of storage stability trials and 87.0% at the end of 20 days. It is possible to say that the polymeric nanomaterial-based sensor system developed in this study is one of the cheapest. The novelties of this study are developing a new prototype device for detecting CA 125 with new polymeric material. As with glucometer devices, the developed method is combined with a portable device, and it is seen as a system that women can apply in their own home environment and have information about their situation. In this way, people who are not sick will be able to check their condition at regular intervals, and patients diagnosed with ovarian cancer will be able to follow up their condition.

## Figures and Tables

**Table 1:** CA 125 analysis with ECLIA and developed method.

ECLIA value (U/mL)	Recovery value (measured value) (U/mL)	% Rec	Error amount (U/mL)	% error in sensor
30	31.703	105.68	+1.703	5.6
50	49.634	99.27	−0.366	0.7
70	69.175	98.82	−0.825	1.18
Standard deviation	0.96			

**Table 2:** Comparison of CA 125 electrochemical analysis methods.

Methods	Materials	Detection range	LOD	Detection time (minutes)	
EIS	NBTB+Nafion modified GCE	0.01–10 ng/mL 50–1000 ng/mL	0.022468 ng/mL	30	[38]
DPV	Au Nanoparticles	-	127 nU/mL	60	[39]
EIS	PAMAM/AuNPs	0.0005–75 U/mL	6 μU/mL	85	[40]
DPV	Poly(3-hydroxyphenylacetic acid) modified SPE	5–80 U/mL	1.45 U/mL	30	[41]
DPV	BN nanosheet modified SPE	5–100U/mL	1.18 U/mL	30	[42]
DPV and CV	Cys-graft-p(HEMA) Nanomaterial	5–400 U/mL	1.87 U/mL	10	This study
